# Professional and Popular Freedom

**Published:** 1915-12-11

**Authors:** 


### The Hospital

The Workers' Newspaper of Administrative Medicine and Institutional Life, Administration, National Insurance and Health.

No. 1539, Vol. LIX. SATURDAY, DECEMBER 11, 1915. PRICE ONE PENNY.

### PROFESSIONAL AND POPULAR FREEDOM.

In a time so strained as that of the present hour the sacrifice of the individual, whether in person or rights or possessions, is a tribute which, in greater or less measure, is imposed upon us all. In some directions the necessity is plain enough, and even from those who respond to no other influence the voice of the tax-gatherer enforces a reply. Outside the area of compulsion advisers are many. Some of them would fain have their suggestions backed by force, others are content with precept, while a third group announce in the newspapers their self-denying example. The circumstances of the day make such proposals appropriate, and no one would lightly check the custom of self-examination out of which they are born. If the advisers are not always agreed among themselves, and if what seems wise to one is condemned as harmful by another, this is a habit which advisers have, and, as we have learned quite recently, even a Coalition Ministry cannot secure unanimity among the experts. That in certain directions we must of necessity be ordered and disciplined for the sake of the national safety, and particularly so in the existing emergency, goes without saying, and this, of course, means that personal inclination or choice must yield to authority. Inevitably in such circumstances authority gains a new prestige, and hence it is not unnatural that some should call for its extension into regions where hitherto individual judgment has enjoyed a large measure of liberty. The process may be seen in operation in various directions, and the medical profession is not escaping such well-intentioned suggestions. As the General Medical Council exists, and as its title in the form here quoted—incorrectly as it happens—appears to propose a supreme authority, the reformers find ready to hand an apparatus by which, could it only be stirred to activity, the numerous wrongs of the situation could be thoroughly and promptly rectified. In our last issue we noted some of the changes which a lay critic would fain impose, and the stream of appeal for authoritative intervention has by no means exhausted itself.

One of the latest demands in this direction is that the medical practitioner shall be periodically re-educated and re-examined with, presumably, as an alternative, the denial of his right to continue to practise. The public, in the shape of a body of patients, may be satisfied with the practitioner's services and may wish to utilise them for its own benefit. But this, it appears, is not enough. At regular intervals there must be a new curriculum and new examinations, and the paterfamilias must, even as his children, learn again to contemplate the dread uncertainty of the inquisition chamber. The public—good, simple souls—must be protected against the "inefficient and presumptuous" by the interrogative habits of wise men clothed with the authority of a governing Council. Their privilege is not to exercise their personal judgment and choice or to utilise their experience and common-sense, but to be "governed" by the greatness and goodness which are invariably found in ministers, councillors, and examiners. If by such a process "inefficiency" could be suppressed and "presumption" rebuked something could be said for the proposal, but seeing that the discipline in various forms has enjoyed a fairly long opportunity without even yet bringing human nature within measurable distance of perfection, we, for our own part, have no faith in its efficacy. Reformers on these lines are always for one more turn of the screw; only grant this and all previous failures will disappear. One compulsory curriculum and one set of examinations leave us still the prey of "the inefficient and presumptuous," but double the pressure and all will be well. It is an ingenuous creed which does credit to the hopefulness of those who profess it. But, whatever be its virtues, we are convinced that we shall never see it in operation. The medical profession is not a very powerful body in the political world, but it will certainly manage to raise trouble on the day when legislative authority shall be sought for a proposal to compel its members to undergo "periodical re-education and examination" at the hands of an examining board. The useful and necessary exercises of youth may not be imposed with impunity on grown men, who must be left to choose their own paths to efficiency. Moreover, all around us is the plain lesson that the public insists upon selecting its own advisers, and that neither examiners nor parchments are accepted as infallible guides. The record of these, truth to tell, at least in this direction, is not without shadow, and those who would multiply them appear too often to forget this. What may be excellent in an appropriate hour becomes merely silly when pressed out of due season.

Still harping on the virtues of authority, the critics call next for power to regulate medical "equipment in all its phases." The ambition is a wide one, and again we think resistance may be expected alike from the profession and the public. The particular lament expressed is that any practitioner can "dub himself a consultant," and can "duly commence work on this much more responsible plane." Apparently it is not recognised that an equal liberty is enjoyed by every British citizen, and "consultants" galore quite unprovided with the wedding garment of a medical diploma abound even in the sacred precincts of Harley Street. The term possesses neither legal protection nor professional value. Even were it provided with these conditions, its restriction, and the restriction of the form of practice it suggests, would invade a liberty which the public would not lightly yield. To deny a patient the right to a further opinion on his case, or to forbid a practitioner to give such an opinion, merely because this latter had not passed a certain examination, is a proposal wholly alien alike to professional traditions and to the public's sense of right and freedom. Consultation practice is earned neither by formal parchments nor by residence in a selected neighbourhood, but by experience and work which command the confidence of the profession. As human judgment is liable to error, this confidence is sometimes misled. But will it inevitably be well guided if the selection of an expert adviser is to be based on some more or less remote achievement certified by examiners who are themselves not beyond the reach of error?

